# Halo-free Phase Contrast Microscopy

**DOI:** 10.1038/srep44034

**Published:** 2017-03-24

**Authors:** Tan H. Nguyen, Mikhail Kandel, Haadi M. Shakir, Catherine Best-Popescu, Jyothi Arikkath, Minh N. Do, Gabriel Popescu

**Affiliations:** 1 Quantitative Light Imaging Laboratory, Department of Electrical and Computer Engineering, Beckman Institute for Advanced Science and Technology, University of Illinois at Urbana-Champaign, Urbana, Illinois 61801, USA; 2 Department of Bioengineering, Micro and Nanotechnology Lab, University of Illinois at Urbana-Champaign, Urbana, Illinois 61801, USA; 3 Munroe-Meyer Institute, University of Nebraska Medical Center (UNMC), Omaha, Nebraska 68198, USA; 4 Computational Imaging Group, Department of Electrical and Computer Engineering, Coordinated Science Lab, University of Illinois at Urbana-Champaign, Urbana, Illinois 61801, USA

## Abstract

We present a new approach for retrieving halo-free phase contrast microscopy (hfPC) images by upgrading the conventional PC microscope with an external interferometric module, which generates sufficient data for reversing the halo artifact. Acquiring four independent intensity images, our approach first measures haloed phase maps of the sample. We solve for the halo-free sample transmission function by using a physical model of the image formation under partial spatial coherence. Using this halo-free sample transmission, we can numerically generate artifact-free PC images. Furthermore, this transmission can be further used to obtain quantitative information about the sample, e.g., the thickness with known refractive indices, dry mass of live cells during their cycles. We tested our hfPC method on various control samples, e.g., beads, pillars and validated its potential for biological investigation by imaging live HeLa cells, red blood cells, and neurons.

Despite its 400-year history, light microscopy continues to be the most common tool in biomedicine^1^. Between the two most important characteristics of a microscopic image: *resolution* and *contrast*, it is improving the latter that has driven most of the technology development in the light microscopy field. Once Abbe described in 1873 *diffraction* as the ultimate limit of the far-field optical resolution, researchers focused mostly on approaching this theoretical limit rather than exceeding it^2^. This dogma remained unchallenged until the 1990’s when the nonlinear optical interaction with the sample proved to be a feasible approach for breaking the diffraction limit with far-field optics^3^.

*Contrast*, on the other hand, has not been proven to be bound by a universal physical law. Unlike the resolution, which is a property entirely of the optical system, the contrast depends on both the instrument and the object of interest (see, e.g., vol. 2 in ref. 4). The main challenge identified early on was to generate images of appreciable contrast when the specimen of interest is transparent. Since such objects, including most live cells, do not absorbs or scatter visible light significantly and because all photo-detectors, including the retina, only respond to power, the resulting intensity distribution across the image is uniform, i.e., the image lacks contrast. This class of transparent specimens is referred to as *phase objects*, pointing to the fact that they only modulate the phase of the incident field and not its amplitude or irradiance (see Chapter 4 in ref. 5).

In the second part of the 19^th^ century, a solution to this problem was developed in the form of tagging the structure of interest with stains or fluorophores, thus, converting the phase specimens into an *amplitude objects*. This approach enjoys the benefit of *specificity*, i.e., the capability of tagging and, thus, imaging only a particular structure of the interest. As a result, these methods of exogenous contrast have become broadly adopted. For example, imaging stained biopsied tissue has been the gold standard in clinical pathology for a century^6^. Also, fluorescence microscopy is the most common form of microscopy in cell biology^7^. However, adding external chemicals to the specimens under investigation is likely to affect its natural structure and function. The fluorescence excitation light, often in the UV range, has been proven toxic to the live cells. In addition to *phototoxicity, photobleaching*, i.e., the irreversible conformational change of a fluorophore that results in fluorescence quenching^8^, is also a major limitation. Photobleaching typically reduces the interval of continuous imaging to only a few minutes.

In response to these challenges, phase contrast (PC) microscopy was developed by Zernike in the 1930’s as a method of intrinsic contrast^9^. Zernike’s idea was founded based on the fundamental understanding of an image as an interferogram, a concept put forward earlier by Abbe^2^. The innovation in PC is as powerful as it is simple: introducing a π/2 phase between the incident and the scattered components of the image field. Suddenly, fine details from within the live, unlabeled cells become visible with high contrast. As a result, PC is now widely used to visualize cells virtually in all biology laboratories. The significant contrast improvement was achieved by treating the microscope as a massively parallel interferometer, in which the incident field acts as the common reference field for all points in the field of view (see Chapter 8 in ref. 10). Clearly, unlike common bright field microscopy, PC requires a spatially coherent illumination field. However, strong spatial filtering necessary to boost the coherence of the light emitted by the microscope lamp comes at the expense of power loss. Thus, current commercial microscopes sacrifice spatial coherence to maintain practical levels of illumination power. As a result, the incident light carries a range of **k**-vectors, instead of just one, which means that the reference of the interference at the image plane is not perfectly flat, but contains spatial structure. The resulting PC image exhibits an artifact, especially at the edges of the object. This artifact, known as the *halo*, has plagued PC ever since its conception, i.e., for more than eight decades.

Here, we present an experimental method that corrects the halo artifact. We experimentally take control over the phase delay between the incident and scattered field. Thus, instead of maintaining a fixed value of π/2 like Zernike’s original system, now, we can tune this phase delay to arbitrary values. As a result, we are able to decouple the amplitude of the interfering fields from their phases. Using these additional data and a new physical optics model that describes quantitatively the halo artifact, we demonstrate that this effect can be reversed by solving an inverse problem. We show halo-free Phase Contrast (hfPC) images of both test samples and biological cells. Furthermore, we show that by acquiring several intensity images at different phase shifts, we can extract quantitatively the path length map associated with the specimen, i.e., the quantitative phase image, free of artifacts. Finally, we demonstrate the application of our approach to study cell growth in large populations.

## Results

### Optical setup

**Figure 1:**
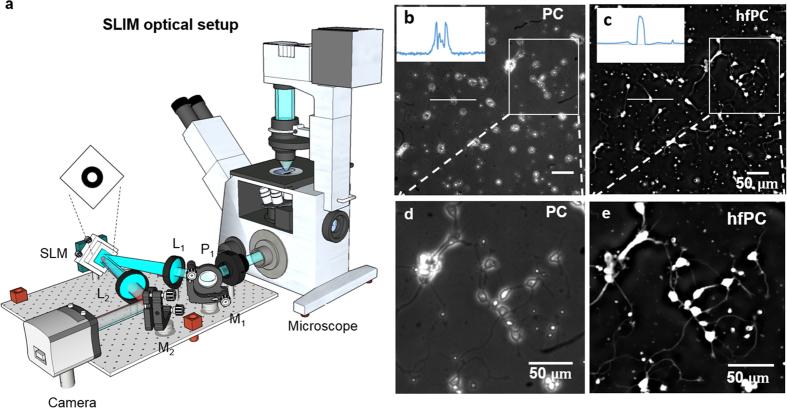
Optical setup. (**a**) The SLIM add-on module uses a 4-f system and a SLM coupled to the output port a phase-contrast microscope. A camera is placed at the output of the module to record the interference intensity. Post-processing is performed on a computer to recover the phase quantity of interest. (**b**) Positive PC image of mouse neurons imaged using a 20x/0.3 NA objective. (**c**) Positive hfPC image. (**d**,**e**) Zoomed-in image of the regions boxed by the rectangle in (**b**) and (**c**) respectively.

Our imaging system consists of a phase-contrast microscope outfitted with an external phase-shifting module. As shown in Fig. 1a, this unit is a re-purposed spatial light interference microscopy (SLIM) module^11^ (CellVista SLIM Pro, Phi Optics, Inc.), designed for quantitative phase imaging^5^. Recently, SLIM has found numerous applications in biomedicine, from cell growth^12^,^13^, tissue scattering^14^,^15^, cancer diagnosis and prognosis^16^,^17^,^18^ and others^19^,^20^,^21^. In SLIM, the total field, *U*_*t*_, emanating from the sample is magnified and replicated in both amplitude and phase at the output port of the microscope. *U*_*t*_ is then polarized by the polarizer P_1_ and Fourier transformed by the lens *L*_1_ at its back focal plane. At this plane, *U*_*t*_is decomposed into the incident field (DC field), *U*_*o*_, and a scattered component (AC field), *U*_*s*_, which are spatially separated. Thus, the spatial Fourier transform of the DC field 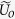 is overlaid with the condenser annulus and the phase ring of the objective. The spatial Fourier transform of the AC component 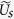 covers the rest of the aperture. A Spatial Light Modulator (SLM), placed at this plane, generates phase-shifting rings to further retard the phase of the DC field in increments of π/2, which becomes 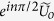, *n* = 0,1,2,3, while leaving the AC field unmodified. Finally, the lens *L*_2_ performs another Fourier transform to form the total field, *U*_*t*_ = *e*^*inπ/2*^*U*_*o*_ + *U*_*s*_, at the camera plane. The camera captures four intensity images, 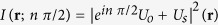, and streams them to a computer to extract phase and amplitude information (Fig 1b–e). Three different objectives with different magnifications, 20x/0.3NA, 40x/0.75NA, and 63x/1.4NA (oil immersed), were used in this work. More details on the optical setup and phase extraction procedure from raw intensity data are shown in the Materials and Methods, Section a.

### Theory

Let *T* be the sample transmission and *U*_*i*_ be the illumination field. The total field emerging from the sample can be written as *U*_*t*_ = *TU*_*t*_. The incident and scattered fields are, respectively, *U*_*o*_ = (*U*_*t*_ ⓥ_r_*h*_*o*_), and *U*_*s*_ = (*U*_*t *_ⓥ_r_*h*_*s*_), with ⓥ_r_ the 2D spatial convolution operator. *h*_*o*_ the Fourier transform of the illumination “ring” pupil, and *h*_*s*_ the Fourier transform of the entire pupil minus the “ring”. The corresponding frequency-domain (transfer) functions are 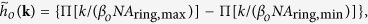 and 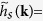 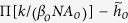, where 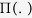 is the disk function defined as 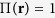 if *r* ≤ 1 and 0, otherwise. *NA*_ring,min_ and 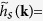
*NA*_ring,max_ are two numerical apertures corresponding to the inner and outer radii of the objective phase ring, *βo* = *ω*_*o*_/*c* is the wavenumber in vacuum, *ω*_*o*_ the angular frequency, and *c* the speed of light in the vacuum. Clearly, 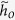 is a band-pass filter, i.e., it retains all spatial frequencies in 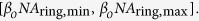 The function 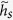 is also a band-pass filter, covering the rest of the spatial bandwidth, i.e., 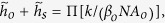 where *NA*_*o*_ is the numerical aperture of the objective. These functions are calculated using experimentally measured parameters of the SLIM system, see the Supplemental Information, Section a. The intensity is given by


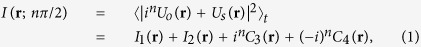


where 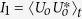, 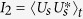, 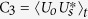, 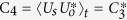. See the Supplemental Information, Section b for a derivation and refs 22 and 23 for more details. *I*_1_, *I*_2_ are the intensities of the incident field and the scattered field, respectively, while *C*_3_ is the temporal cross-correlation function of the two fields at zero temporal delays, *τ* = 0^24^. The closed-form formulas for these quantities under partially coherent illumination are given in the Supplemental Information, Section b. By the Cauchy-Schwarz inequality for inner products, we have *C*_3_*C*_4_ ≤ *I*_1_*I*_2_. An equality holds in the temporal domain when 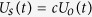, ∀*t* or 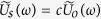, ∀*ω*, the optical frequency. Combining with the definitions of *U*_*o*_ and *U*_*s*_, we have 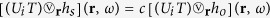, which is satisfied when the filtering operations are not dependent on the optical frequency *ω*, i.e., *h*_*s*_(**r**, *ω*) = *h*_*s*_(**r**) and *h*_*o*_(**r**, *ω*) = *h*_*o*_(**r**). Assuming this condition is satisfied, *C*_3_*C*_4_ = *I*_1_*I*_2_, and using the four intensity images, one can solve for *I*_1_, *I*_2_, *C*_3_, *C*_4_ explicitly (See Supplemental Information, Section b). Summing the solutions for *I*_1_ and *C*_4_ gives (see Section c of the Supplemental Information for a derivation)


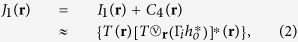


where


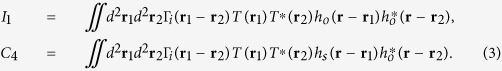


See ref. 22 and Section b of the Supplemental Information for the derivation. In Eq. 3, Γ_*i*_ is the mutual intensity of the illumination at the sample plane. Our goal is to solve for the sample transmission *T* when *J*_1_ is known. For a phase object with transmission *T*(**r**) = e^*iϕ*(r)^ taking the arguments on both sides of Eq. (2), we obtain the expression for the experimentally measured phase as


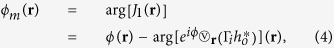


Clearly, the effects of phase underestimation and “halo effect”, which are well-known in phase contrast microscopy^9^,^25^, can be seen directly from Eq. (4). A discussion of these phenomena can be found in the Supplemental Information. To obtain the correct estimation *ϕ*^†^(**r**) of *ϕ*(**r**) from the measured signal *J*_1_(**r**), we solve the following constrained optimization problem optimization problem


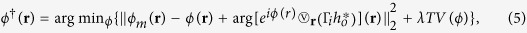


In Eq. 5, 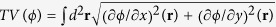, is the Total Variational term^26^,^27^, which suppresses the effect of noise and enforces the sparsity assumption on the gradient of the reconstruction. ||.||_2_ denotes the *l*_*2*_-norm. The parameter *λ* is a trade-off factor that balances the measurement error and the TV term. The problem is solved using the limited-memory Broyden-Fletcher-Goldfarb-Shanno algorithm with *box* constraints (L-BFGS-B)^28^,^29^,^30^, enforcing the solved phase to satisfy the non-negative constraint at each pixel, i.e., *ϕ*(**r**) ≥ 0. More details on the source code and post-processing steps can be found in Supplemental Information, Section e. With this inversion, we can then numerically reconstruct both halo-free phase contrast images (hfPC) as well as halo-free quantitative phase images (hfQPI).

### Halo-free phase contrast images of neurons

Using this approach, we illustrate the halo removal in Zernike’s phase contrast images of mouse neurons. The sample preparation protocol can be found in Materials and Methods. Figure 1b,c show positive and negative phase contrast images of neurons images using a 20x/0.3 NA objective. From the four QPI images, the halo-free phase map *ϕ*^†^ is obtained by solving Eq. (5) used to calculate the halo-free phase contrast (PC) image (see the Supplemental Information, Section f, for details). These halo-free PC (hfPC) images are equivalent to what it would be measured with an infinitely thin ring of illumination. Images of zoomed-in regions from Fig. 1b,c are shown in Fig. 1d,e, respectively. A formation of a neural network can be observed in this region from the halo-free positive phase contrast image, without the typical artifacts associated with phase contrast. The line profiles in Fig. 1b,c show how the negative values commonly associated with halos disappeared as a result of our method.

### Thickness measurement of nanoscale topography samples

**Figure 2:**
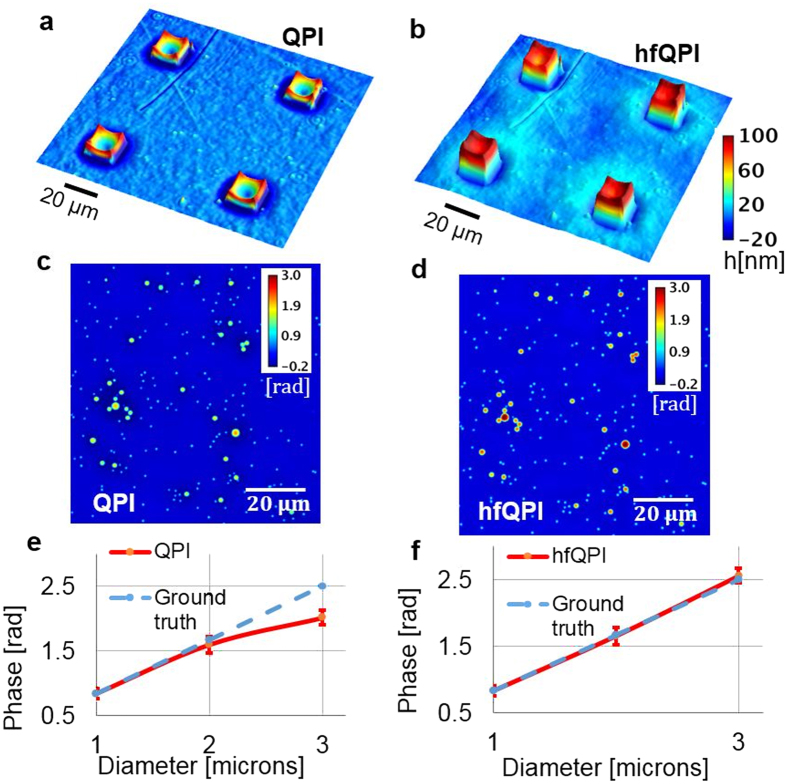
Halo removal of square micro-pillars and polystyrene beads. (**a**) Input height surface map of four quartz micro-pillars that are 20-μm wide and 80-nm high measured using a 20x/0.3 NA objective. The unit is nm. (**b**) The halo-free surface map of (**a**). (**c**) Measured phase map of a mixture of 1, 2 and 3-μm polystyrene beads image under the same setup. Note that the halo affects less the small beads than to the large beads. (**d**) Halo-free version of (**c**). (**e**,**f**) Diameter profiles for different sizes of the beads for (**c**) and (**d**) respectively. Dashed lines are expected ground truth profiles.

The hfQPI can be used to provide highly accurate topography measurements at the nanoscale. In order to demonstrate that halo-free images can be used to profile quantitatively transparent samples, we measured the thickness of quartz micropillar samples. The pillars are square, 10, 20 and 40-μm wide and 80-nm thick, as verified by the Alpha-Step IQ Profilometer. Figure 2a shows the thickness profile measured by QPI using a 20x/0.3 NA phase contrast objective of 20-μm wide pillars. The thickness is obtained from the phase measurement *ϕ*_*m*_ using 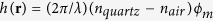, where the refractive indices of quartz, *n*_*quartz*_ = 1.545 and *n*_*air*_ = 1 at a mean wavelength of *λ* = 547 nm. The hfQPI image, *ϕ*^†^(**r**), is obtained using Eq. (5) and converted into the thickness map as shown in Fig. 2b. It can be seen that the negative thickness area surrounding the pillars in Fig. 2a is eliminated in Fig. 2b. Also, the thickness of the reconstructed pillars converges to our expected value of 80 nm. To test the limits of our method, we applied it to a variety of pillar sizes, using different magnifications. The performance of the reconstruction algorithm is characterized by dividing the area under the height profile through the center of the pillar to the expected area under perfect reconstruction, i.e., without the halo (see Supplemental Information, Section g).

We validated our method further by removing the halo from images of a mixture of polystyrene microbeads (Polysciences Inc) with 3 different diameters (1 μm, 2 μm, 2 μm)±5%, of refractive index 1.59. The beads were mixed in ethanol before being dispersed onto the surface of a cover glass and exposed to air for drying out for 15 minutes. At that point, immersion oil (Zeiss) with a refractive index value of 1.518 was applied and a cover slip placed on top of the oil droplet to flatten it. Figure 2c,d show the original QPI phase map of the beads and the reconstructed phase map, respectively. From these images, for each diameter, the mean and standard deviation of the phase are extracted from 5 beads in the field of view. The phase values are then compared with the theoretical phase of 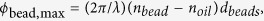 where *d*_*beads*_ are their known diameters. The measured vs. calculated phase values are displayed in Fig. 2e,f. In Fig. 2e, we show the measured phase values obtained from the original QPI image, while Fig. 2f shows the hfQPI image. It can be seen that the hfQPI phase matches very well the expected phase values across all dimensions of the beads.

### hfPC of biological samples

**Figure 3:**
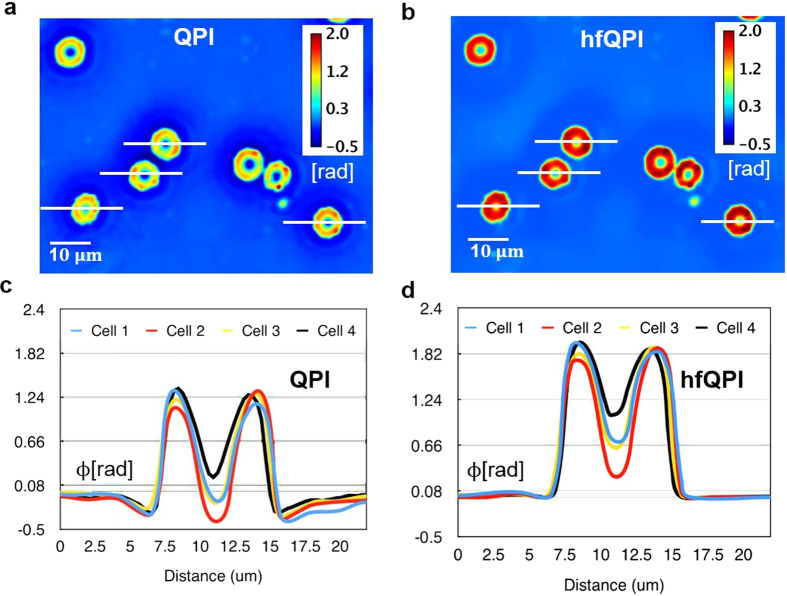
Halo removal of red blood cells (**a**,**b**) Original QPI image and hfQPI image version of the same red blood cell sample measured under 40x/0.75 NA. (**c**,**d**) Phase profiles of several red-blood cells selected in (**a**) and (**b**) respectively.

We further tested our method on red blood cells. Figure 3a shows the original QPI image and Fig. 3b are the hfQPI image. Their phase cross-sections through 4 different cells are illustrated in Fig. 3c,d, respectively. Using *n*_*PBS*_ = 1.334, *n*_*hemoglobin*_ = 1.402^31^, the phase values of 1.82 ± 0.14 radians from profiles in Fig. 3(d) estimate the thickness of these cells to be 2.2 ± 0.17 μm, which falls within the normally expected range^32^.

**Figure 4:**
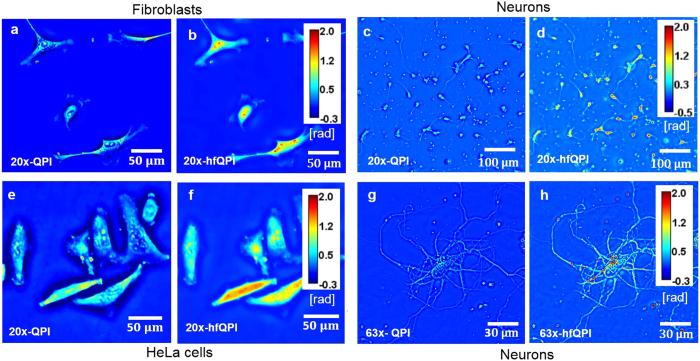
Original QPI and hfQPI images of different samples at different magnifications, as indicated.

Our halo correction method is applicable to a broad range of specimens, objective numerical apertures, and magnifications. For example, Fig. 4a,b show fibroblasts and Fig. 4c,d display the neurons imaged at 20x/0.65 NA, respectively. Figure 4e,f shows HeLa cells at 40x/0.75 NA and Fig. 4g,h are the neurons at 63x/1.4 NA. For all cases, the halo is suppressed while maintaining the details in the original QPI, e.g., cell bodies, dendrites, axons and their terminals, etc., which indicates that our method is scale invariant and universally applicable. The effects of halo correction can be accessed quantitatively by investigating the histogram of phase values (see the Supplemental Information, Section h).

### hfQPI imaging of cell populations

**Figure 5:**
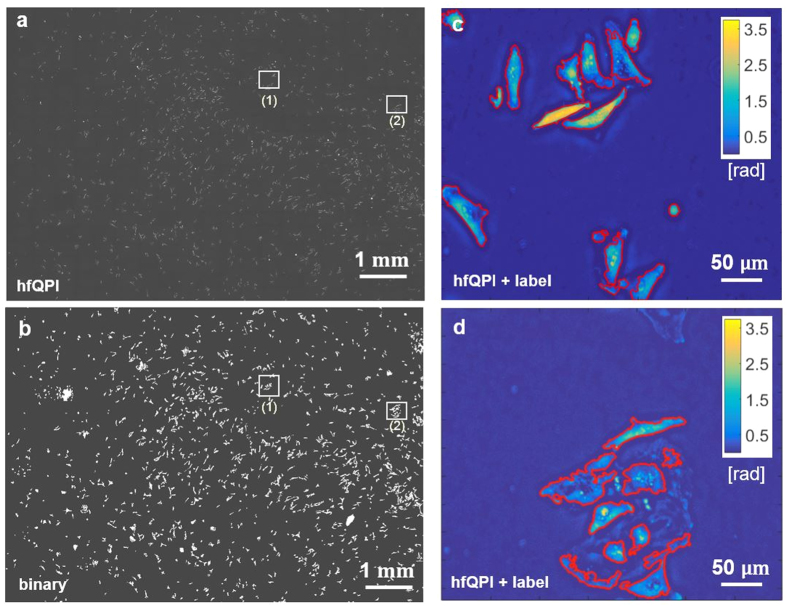
Automatic cell segmentation. (**a**) Stitching results of hfQPI images over a large FOV. (**b**) Stitching results of automatic binary segmentation. (**c,d**) Segmentation results overlaid on the hfQPI images of zoomed-in regions (1) & (2) in (**a**) and (**b**) respectively.

Based on the quantitative phase information, we can successfully analyze entire cell populations and study their growth. With the halo artifact suppressed, automatic cell segmentation can be performed very efficiently. In the past, often image segmentation methods that relied on thresholding of the local intensity were confused by the negative values of the halo^33^,^34^,^35^. We show that hfQPI images can offer very accurate segmentation results for HeLa cells with only a few processing steps required. We imaged a 30% confluence HeLa cell culture over 33 hours. A large field of view (FOV), 10.875 × 8.125 mm^2^, consisting of 25 × 25 individual frames (1392 × 1040 pixels each), was imaged with a 20×/0.3 NA objective and a spatial sampling of 3.2 pixels/μm. Figure 5a shows stitched images of all HeLa at the first time step *t* = 0 minutes. The halo-removal procedure was applied to all images to obtain the corresponding hfQPI phase maps. The cells were segmented from the hfQPI as described in the Supplemental Information, Section i. It takes approximately 1.5 hours to segment the cells in all the 65,000 frames. All binary maps resulting from the segmentation are stitched together for each time step and shown in Fig. 5b. Figure 5c,d show cell boundaries overlaid on the hfQPI images for the two regions indicated by the boxes in Fig. 5a,b, respectively. It can be seen clearly that automatically detected cell boundaries align very well with the true boundaries of the cells from the phase image.

### Cell growth study using hfQPI images

**Figure 6:**
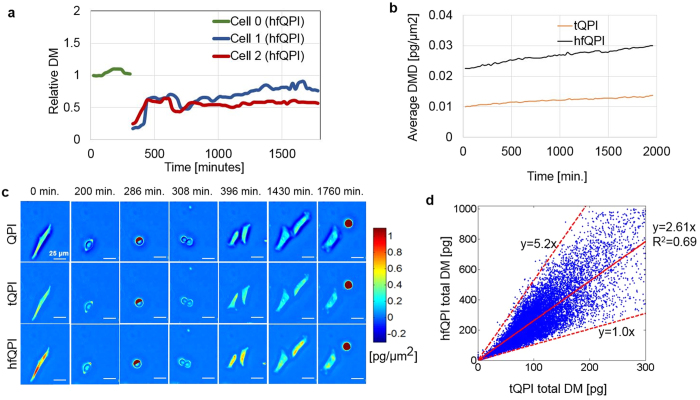
HeLa cell mass measurement. (**a**) Different growth curves from a parent and two daughter HeLa cells measured using a 20x/0.3 NA objective over 32.6 hours. Each growth curves show the total dry mass of a single HeLa cell over time. When a parent cell divides, two new curves are generated for the daughter cells. (**b**) Measured averaged dry mass densities obtained from the thresholded quantitative phase imaging (tQPI) images and the hfQPI images over time. (**c**) Each row shows seven measurements of the dry mass density at different time points in (**a**). The first row contains the raw QPI images. The middle row shows tQPI images. The bottom row is for hfQPI images. (**d**) Scatter plot of the total dry mass of all several cells obtained from tQPI images (horizontal axis) and the hfQPI images (vertical axis) using automatic segmentation. The lines show the maximum slope, minimum slope and fitted slope using linear regression relations between these two quantities.

In his seminal paper^36^, Barer established that the dry mass density *ρ*(**r**) is proportional to the optical phase *ϕ*, following the relation *ρ* = *λϕ*/(2*πγ*), where *λ* is the central wavelength of the illumination and *γ* ≈ 0.2*ml/g* is the refractive index increment. We used this relation to determine the total dry mass of each single cell by integrating over its area. Figure 6a shows the relative dry mass of a HeLa parent cells (green curve) and that of its two daughter cells (red and blue curves) over 35 hours with a time step of 32 minutes.

The dry mass values are normalized to the initial mass of the parent cell at time *t* = 0 minutes to obtain the relative dry mass change. These data are obtained from raw dry mass after being smoothed over time using a window size of three frames. Figure 6b shows the average dry mass densities computed over the full field of view as a function of time. This quantity is obtained by dividing the total mass of all cells by the total area covered, for each time point. The red profile is the average dry mass density calculated using raw QPI images thresholding out the negative phase values while the blue one is obtained using the hfQPI images. It can be seen that the ratio of these two densities is approximately 2.5 throughout the entire time lapse. Figure 6c illustrates original QPI images, thresholded QPI (tQPI) images and hfQPI images of these cells at different time points. The correlation between the dry mass obtained from tQPI and hfQPI images is shown in Fig. 6d. Each point in this scatter plot corresponds to a single cell at one time-step. The horizontal coordinate is the total dry mass from the tQPI images while the vertical one is the total dry mass from the halo-free images. All dry mass values below 25 pg and above 500 pg from the tQPI images are eliminated from the analysis to reduce errors due to debris. It can be seen that the dry mass obtained from hfQPI and tQPI images can be described by a linear relation 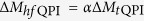 with 1.0 ≤ *α* ≤ 5.2 and a standard deviation value of 0.82. The reason for *α* not being a constant can be explained using Fig. 6c. Each column of this figure corresponds to a different time in a cell cycle. It can be seen that the effect of the halo is not the same at all times. While the halo can be seen clearly when the cell is in interphase (e.g. *t* = 0 minute, *t* = 396 minutes, and *t* = 430 minutes), the halo almost disappears when the cell balls up during mitosis (*t* = 1760 minutes). The halo-removal correction works on all of these cases. However, the amount of correction applied to cells in the interphase is larger than that in mitosis. For *t* = 1760 minutes, the mitotic HeLa cells before and after correction are very similar to each other. Therefore, the total dry mass from the halo-free QPI image and that from the tQPI image will have *α* ≈ 1. On the contrary, in column 1 of Fig. 6c, the corrected QPI image gives a phases value approximately twice those in the tQPI image. As a result, this case will give a ratio *α* ≥ 2. Using linear regression fitting, we obtain *α* = 2.61 ± 0.82 and the *R*^2^ coefficient of 0.69. This fitting slope only varies slightly over different regions. For example, *α* = 2.53 ± 0.81, 2.64 ± 0.83, 2.69 ± 0.80, when the original mass from tQPI is in [0,100], [100,200], [200,300] pg, respectively.

**Figure 7:**
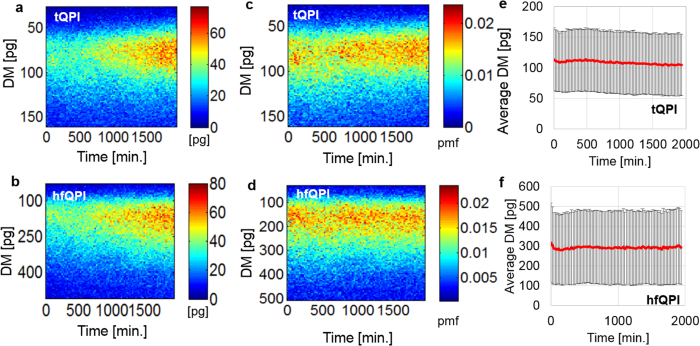
(**a**,**b**) Total dry mass histogram of all cells in the FOV obtained from the tQPI and hfQPI images over time, respectively. Each column corresponds to one time-step. Each row corresponds to bin of the dry mass histogram. (**c**,**d**) Normalized version of (**a**) and (**b**) to the number of cells, respectively. Therefore, they show the probability mass function of a single cell. (**e**,**f**) The mean and the standard deviation of the mass over time obtained from tQPI and hfQPI images, respectively. DM: single cell dry mass.

Finally, we analyzed the dry mass distribution of each cell. Figure 7a,b show dry mass histograms over 88 time points (one column per time point) for all cells. The cells were segmented automatically. All regions with areas less than 5,000 pixels (488.28μm^2^) are excluded from the analysis to lower the error due to of small debris. Figure 7a is obtained from tQPI images, while Fig. 7b is from hfQPI images. Each histogram consists of 200 bins. It can be seen that, over time, the total number of counts increases due to continuous cell division. The cells become more diverse in dry mass, resulting in a broadening of the histogram distribution toward the areas of larger and smaller dry masses. Figure 7c,d are normalized versions of Fig. 7a,b, respectively, to the number of cells at each time point. Therefore, they are equivalent to the probability mass function of the single cell dry mass at each time. The mean and standard deviation of the cell dry mass of Fig. 7c,d are shown in Fig. 7e,f, respectively. It can be seen that although there is more variation in the population, the average values of the dry mass only slightly reduces for the tQPI and essentially stays constant for the hfQPI over time. The standard deviation values are approximately the same over time for both cases.

The studies on cells reported in this paper have been performed in accordance with the protocols approved by the Institutional Review Board at the University of Illinois at Urbana-Champaign (IRB Protocol Number: 13900).

## Discussion

We introduced a new method to remove the halo artifact from the classical phase contrast microscopy. Our method combines highly sensitive light interferometry, theoretical optics, and computational algorithms. Our approach is able not only to correct for this artifact but also to produce quantitatively the phase value introduced by the object, which is valuable in studying various problems, such as nanoscale profilometry of materials and quantitative cell biology. We tested our method on various control nanofabricated samples as well as biological samples e.g. neurons, red blood cells, and HeLa cells under different magnifications. Our results showed that the dry mass of HeLa cells can be obtained directly from hfQPI images without the need to scan through the full volume of the cell. By scanning very large fields of view over a period of 35 hours and combining with an algorithm for automatic cell segmentation, the dry mass density distribution for the whole population was obtained easily and quickly. Interestingly, we found that the relative dry mass obtained directly from tQPI images and that from the hfQPI images are strongly correlated with a linear proportionality constant of 2.6. Therefore, one can obtain a good estimate for the growth curve directly from tQPI images. However, for high accuracy, hfQPI must be used instead. We anticipate that various studies of cell functions using phase contrast microscopy can benefit from these results.

As a label-free method, PC microscopy can be applied to imaging live cells nondestructively over broad time scales. This ability is not limited by photobleaching and phototoxicity commonly associated with fluorescence microscopy. At the same time, PC lacks specificity. Therefore, we envision that PC and fluorescence techniques will co-exist and corroborate the advantages of specificity and noninvasiveness. It is particularly valuable that our optical system operates on the same optical path as the fluorescence channels, which makes combining the two channels very practical. Although applied here to monolayers of cells, the halo-removal approach is expected to work without modifications in imaging thin tissue slices, relevant to histology.

## Methods

### Imaging

We used a SLIM module (CellVista SLIM Pro, Phi Optics Inc.) coupled to an inverted phase contrast microscope (Axio Observer Z1, Zeiss Inc.), under white-light illumination. Three different phase contrast objectives were used: LD Achroplan 20x/0.40 NA Korr Ph2 (Part number: 440845-0000-000) objective, the EC Plan-Neofluar 40x/0.75 NA Ph2 (Part number: 420361-9910-000) one, and the Plan-Apochromat 63x/1.4 NA Oil Ph3 one (Part number: 420781-9910-0000). This QPI module uses a Meadowlark 512 × 512 high-speed reflective SLM placed at the conjugate plane to the back focal plane of the objective. The SLM operates in phase mode with incident light linearly polarized by the polarizer *P*_1_. This polarizer is placed at a plane conjugated to the sample plane in order to minimize optical aberration. The output port of the QPI module is equipped with an Andor’s Zyla 5.5 megapixel sCMOS camera, able to capture images at the maximum frame rate of 100 fps. Four modulating patterns on the SLM are alternatively applied with an SLM delay of 10 ms, giving effective framerate up to 15 fps for QPI imaging.

### Sample preparations

#### Micro-pillars

The micro-pillars were prepared from a 1”-quartz wafer patterned using SPR 511a positive photoresist and transferred to the quartz substrate by etching in a reactive-ion-etcher (RIE) using a CF4 (Freon 14 plasma).

#### Red blood cells

The red blood cells were obtained from our local hospital using venipuncture and stored in a refrigerator at 4 °C. Informed consent was obtained from all subjects. The blood was diluted to a concentration of 0.2% in PBSA solution (0.5% Bovine Serum Albumin in PBS). To prevent cell tilt during imaging, a sample chamber was prepared by punching a hole into a piece of double-sided scotch tape and sticking the tape onto a coverslip. After dispensing a drop of blood into this circular chamber, the drop was sealed from the top by a Poly-L-lysine coated coverslip. The coverslip pair was then turned over and the cells were allowed to settle for 1 hour before imaging so that they become immobilized.

#### Neurons

Cortical neurons, obtained from the referenced source, were cultured and maintained from postnatal (P0-P1) C57BL/6 mice as previously described^37^. All animal procedures were carried out in accordance with approved protocols from the Institutional Animal Care and Use Committees (IACUC) at University of Nebraska Medical Center and University of Illinois Urbana Champaign, and in tight accordance with the recommendations in the Guide for the Care and Use of Laboratory Animals of the National Institutes of Health. (Animal Assurance PHS: #A3294-01, Protocol Number: 10-033-08-EP). Aliquots of frozen neurons were rapidly thawed at 37 °C and plated on poly-D-lysine-coated 35 mm glass petri^38^. Low-density cultures (65 cells per mm^2^) were grown at 37 °C, in the presence of 5% CO_2_, in standard maintenance media containing Neurobasal growth medium supplemented with B-27 (Invitrogen), 1% 200 mM glutamine (Invitrogen) and 1% penicillin/streptomycin (Invitrogen). Half the media was aspirated twice a week and replaced with fresh maintenance media warmed to 37 °C. Live imaging studies took place on DIV 3.

## Additional Information

**How to cite this article:** Nguyen, T. H. *et al*. Halo-free Phase Contrast Microscopy. *Sci. Rep.* **7**, 44034; doi: 10.1038/srep44034 (2017).

**Publisher's note:** Springer Nature remains neutral with regard to jurisdictional claims in published maps and institutional affiliations.

## Supplementary Material

Supplementary Information
