# Supplementary material for: Halo-free Phase Contrast Microscopy
Source: Sci Rep. 2017 Mar 24;7:44034. doi: 10.1038/srep44034 (PMC5364506; doi:10.1038/srep44034)
Supplement: Supplementary Information [file srep44034-s1.docx]

**Supplemental Information:** **Halo-free Phase Contrast Microscopy**

Tan H. Nguyen^1†^, Mikhail Kandel^1^, Haadi M. Shakir^2^, Catherine Best-Popescu^2^, Jyothi Arikkath^3^, Minh N. Do^4^, and Gabriel Popescu^1*^

*e-mail: [gpopescu@illinois.edu](mailto:gpopescu@illinois.edu)

1. **Characterizing** $\Gamma_{i},h_{o},h_{s}.$

For a successful inversion, the functions $\Gamma_{i},$ $h_{o},$ and $h_{s}$ need to be generated with high accuracy. Again, $\Gamma_{i}$ is the mutual intensity function at the sample plane. $h_{o}$ and $h_{s}$ are the point spread functions (PSFs) corresponding to the ring and non-ring regions of the objective, respectively. These functions are characterized by the illumination wavelength, the numerical aperture of the objective,$NA_{o},$ and the illumination aperture, governed by$NA_{\text{ring,min}}$ and $NA_{\text{ring,max}}$. While $NA_{o}$ is provided by the microscope manufacturer, other parameters are not always available. In order to determine them, we imaged the back aperture of the objective onto the camera plane so that the inner radius, $r_{\text{ring,in}},$ outer radius, $r_{\text{ring,out}},$ of the phase ring and that of the objective’s aperture, $r_{o},$ can be measured experimentally. With these radii available, the inner and outer numerical apertures of the phase ring are determined using following relations $NA_{\text{ring,min}}=r_{\text{ring,in}}NA_{o}/r_{o},$ $NA_{\text{ring,max}}=r_{\text{ring,out}}NA_{o}/r_{o}.$ Also, in our setup, a phase contrast objective was used. The phase ring of this objective has an attenuation factor to reduce the amplitude of the incident field, hence, maximizing the contrast of the PC. This factor can be accounted by manipulating the kernel $h_{o}$ given that its value is accurately measured. To determine its value, we calculate the ratio between the average intensity over a line profile inside and outside the objective phase ring. Finally, the function $\Gamma_{i}$ is obtained by two-dimensional Fourier transforming the intensity of the condenser aperture, i.e., $\Gamma_{i}\left(\Delta\mathbf{r}\right)=\Im\left[I_{c}\left(\mathbf{k}\right)\right]\left(\Delta\mathbf{r}\right)$, where $\Im[.]$ denotes the the spatial Fourier transform operator.

1. **Solving for** $I_{1},I_{2},C_{3},C_{4}.$

Equation (1) in the main text reads

$I\left(\mathbf{r};n\pi/2\right)=I_{1}+I_{2}+i^{n}C_{3}+\left(-i\right)^{n}C_{4},$
where four individual terms $I_{1},I_{2},I_{3},I_{4}$ are defined by

$I_{1}=\iint d^{2}\mathbf{r}_{1}d^{2}\mathbf{r}_{2}\Gamma_{i}\left(\mathbf{r}_{1}-\mathbf{r}_{2}\right)T\left(\mathbf{r}_{1}\right)T^{*}\left(\mathbf{r}_{2}\right)h_{o}\left(\mathbf{r}-\mathbf{r}_{1}\right)h_{o}^{*}\left(\mathbf{r}-\mathbf{r}_{2}\right),$ (2a)

$I_{2}=\iint d^{2}\mathbf{r}_{1}d^{2}\mathbf{r}_{2}\Gamma_{i}\left(\mathbf{r}_{1}-\mathbf{r}_{2}\right)T\left(\mathbf{r}_{1}\right)T^{*}\left(\mathbf{r}_{2}\right)h_{s}\left(\mathbf{r}-\mathbf{r}_{1}\right)h_{s}^{*}\left(\mathbf{r}-\mathbf{r}_{2}\right),$ (2b)

$C_{3}=\iint d^{2}\mathbf{r}_{1}d^{2}\mathbf{r}_{2}\Gamma_{i}\left(\mathbf{r}_{1}-\mathbf{r}_{2}\right)T\left(\mathbf{r}_{1}\right)T^{*}\left(\mathbf{r}_{2}\right)h_{o}\left(\mathbf{r}-\mathbf{r}_{1}\right)h_{s}^{*}\left(\mathbf{r}-\mathbf{r}_{2}\right),$ (2c)

$C_{4}=\iint d^{2}\mathbf{r}_{1}d^{2}\mathbf{r}_{2}\Gamma_{i}\left(\mathbf{r}_{1}-\mathbf{r}_{2}\right)T\left(\mathbf{r}_{1}\right)T^{*}\left(\mathbf{r}_{2}\right)h_{s}\left(\mathbf{r}-\mathbf{r}_{1}\right)h_{o}^{*}\left(\mathbf{r}-\mathbf{r}_{2}\right).$ (2d)

Equations 2a-d are generally valid under partially coherent illumination, governed by the mutual intensity $\Gamma_{i}$. To prove Eqs. 2a-d, let us start by considering that the point sources at the condenser plane are independent of each other. Each point source in the condenser aperture, which is characterized by a transverse spatial frequency of $\mathbf{k},$ generates a plane wave $A_{c}\left(\mathbf{k}\right)e^{i\mathbf{kr}}$onto the sample plane, yielding a new total field of $\left(e^{i\mathbf{k}.\mathbf{r}}T\right)$ right after it. The unmodulated region of the back aperture, i.e. the non-ring one, with the PSF of $h_{s}$ generate a coherent response of $\left(e^{i\mathbf{k}.\mathbf{r}}T\right)ⓥ_{\mathbf{r}}h_{s}$. The modulating region with the phase modulation of $n\pi/2$ alters the PSF to $i^{n}h_{o}$ and give a coherent response at the camera plane of $\left(e^{i\mathbf{k}.\mathbf{r}}T\right)ⓥ_{\mathbf{r}}\left(i^{n}h_{o}\right).$ Combining the responses with a contribution of an intensity term of $I_{c}\left(\mathbf{k}\right)=A_{c}^{2}\left(\mathbf{k}\right)$ for different wave vectors $\mathbf{k},$we have the total intensity image of [^1^](#_ENREF_1)

$$I\left(\mathbf{r};n\pi/2\right)=\int d^{2}\mathbf{k}I_{c}\left(\mathbf{k}\right)\left|\left[\left(e^{i\mathbf{k}.\mathbf{r}}T\right)ⓥ_{\mathbf{r}}\left(h_{s}+i^{n}h_{o}\right)\right]\right|^{2}\left(\mathbf{r}\right).$$

Expanding the convolution operation, , we further obtain

$$\begin{matrix} I\left(\mathbf{r};n\pi/2\right)=\iint d^{2}\mathbf{r}_{1}d^{2}\mathbf{r}_{2}\left[d^{2}\mathbf{k}I_{c}\left(\mathbf{k}\right)e^{i\mathbf{k}.\left(\mathbf{r}_{1}-\mathbf{r}_{2}\right)}\right]T\left(\mathbf{r}_{1}\right)T^{*}\left(\mathbf{r}_{2}\right)h_{o}\left(\mathbf{r}-\mathbf{r}_{1}\right)h_{o}^{*}\left(\mathbf{r}-\mathbf{r}_{2}\right) \\ +\iint d^{2}\mathbf{r}_{1}d^{2}\mathbf{r}_{2}\left[d^{2}\mathbf{k}I_{c}\left(\mathbf{k}\right)e^{i\mathbf{k}.\left(\mathbf{r}_{1}-\mathbf{r}_{2}\right)}\right]T\left(\mathbf{r}_{1}\right)T^{*}\left(\mathbf{r}_{2}\right)h_{s}\left(\mathbf{r}-\mathbf{r}_{1}\right)h_{s}^{*}\left(\mathbf{r}-\mathbf{r}_{2}\right) \\ +\left(-i\right)^{n}\iint d^{2}\mathbf{r}_{1}d^{2}\mathbf{r}_{2}\left[d^{2}\mathbf{k}I_{c}\left(\mathbf{k}\right)e^{i\mathbf{k}.\left(\mathbf{r}_{1}-\mathbf{r}_{2}\right)}\right]T\left(\mathbf{r}_{1}\right)T^{*}\left(\mathbf{r}_{2}\right)h_{s}\left(\mathbf{r}-\mathbf{r}_{1}\right)h_{o}^{*}\left(\mathbf{r}-\mathbf{r}_{2}\right) \\ +i^{n}\iint d^{2}\mathbf{r}_{1}d^{2}\mathbf{r}_{2}\left[d^{2}\mathbf{k}I_{c}\left(\mathbf{k}\right)e^{i\mathbf{k}.\left(\mathbf{r}_{1}-\mathbf{r}_{2}\right)}\right]T\left(\mathbf{r}_{1}\right)T^{*}\left(\mathbf{r}_{2}\right)h_{o}\left(\mathbf{r}-\mathbf{r}_{1}\right)h_{s}^{*}\left(\mathbf{r}-\mathbf{r}_{2}\right). \end{matrix}$$

Finally, using the Fourier relation between $\Gamma_{i}\left(.\right)$, the mutual intensity of the illumination, and the aperture intensity[^2^](#_ENREF_2), i.e., $\Gamma_{i}\left(\Delta\mathbf{r}\right)=\Im\left[I_{c}\left(\mathbf{k}\right)\right]\left(\Delta\mathbf{r}\right)$, Eq. becomes

$$\begin{matrix} I\left(\mathbf{r};n\pi/2\right)=\iint d^{2}\mathbf{r}_{1}d^{2}\mathbf{r}_{2}\Gamma_{i}\left(\mathbf{r}_{1}-\mathbf{r}_{2}\right)T\left(\mathbf{r}_{1}\right)T^{*}\left(\mathbf{r}_{2}\right)h_{o}\left(\mathbf{r}-\mathbf{r}_{1}\right)h_{o}^{*}\left(\mathbf{r}-\mathbf{r}_{2}\right) \\ +\iint d^{2}\mathbf{r}_{1}d^{2}\mathbf{r}_{2}\Gamma_{i}\left(\mathbf{r}_{1}-\mathbf{r}_{2}\right)T\left(\mathbf{r}_{1}\right)T^{*}\left(\mathbf{r}_{2}\right)h_{s}\left(\mathbf{r}-\mathbf{r}_{1}\right)h_{s}^{*}\left(\mathbf{r}-\mathbf{r}_{2}\right) \\ +\left(-i\right)^{n}\iint d^{2}\mathbf{r}_{1}d^{2}\mathbf{r}_{2}\Gamma_{i}\left(\mathbf{r}_{1}-\mathbf{r}_{2}\right)T\left(\mathbf{r}_{1}\right)T^{*}\left(\mathbf{r}_{2}\right)h_{s}\left(\mathbf{r}-\mathbf{r}_{1}\right)h_{o}^{*}\left(\mathbf{r}-\mathbf{r}_{2}\right) \\ +i^{n}\iint d^{2}\mathbf{r}_{1}d^{2}\mathbf{r}_{2}\Gamma_{i}\left(\mathbf{r}_{1}-\mathbf{r}_{2}\right)T\left(\mathbf{r}_{1}\right)T^{*}\left(\mathbf{r}_{2}\right)h_{o}\left(\mathbf{r}-\mathbf{r}_{1}\right)h_{s}^{*}\left(\mathbf{r}-\mathbf{r}_{2}\right), \end{matrix}$$

Thus, Eqs. 1-2 follow by identifying the four terms in Eq. . Although this equation has been used intensively to study the image formation under partially coherent illumination [^1^](#_ENREF_1),[^3^](#_ENREF_3), it is rarely used in solving the inverse problem to recover the sample transmission, $T$, due to high computational complexity. For example, consider a transmission map $T$ of $N\times N$ pixels and all kernels $\Gamma_{i},h_{o},h_{s}$of $P\times P$ pixels, computing intensity $I$ requires $O\left(N^{2}P^{4}\right)$ operations, which would be problematic for large values of $N$ and $P$.

Although we have four unknowns with four intensity measurements $I\left(\mathbf{r};n\pi/2\right)$ with $n=0,1,2,3,$ there is still an ambiguity in resolving $I_{1}$ and $I_{2}$ since the same combination $I_{1}+I_{2}$ appears in all terms. Note that $C_{3}$ and $C_{4}$ are conjugated to one another. Solving only for one of them is sufficient. $C_{3}$ can be obtained precisely from 4 frames as

$$C_{3}\left(\mathbf{r}\right)=\left(1/2\right)\left\{\left[I\left(\mathbf{r};0\right)-I\left(\mathbf{r};\pi\right)\right]+i\left[I\left(\mathbf{r};-\pi/2\right)-I\left(\mathbf{r};\pi/2\right)\right]\right\}.$$

An extra equation is needed to resolve $I_{1}$ and $I_{2}$. Toward this end, we assume the illumination to be close to spatially coherent, or that the field is quasi-plane wave, analog to quasi-monochromatic in the temporal domain. With this approximation, we obtain, [^4^](#_ENREF_4)

$$\begin{matrix} I_{1}I_{2}\approx C_{3}C_{4}=\left(1/4\right)\sqrt{\left[I\left(\mathbf{r};0\right)-I\left(\mathbf{r};\pi\right)\right]^{2}+\left[I\left(\mathbf{r};-\pi/2\right)-I\left(\mathbf{r};\pi/2\right)\right]^{2}} \\ \equiv P. \end{matrix}$$

The sum $S$ of $I_{1}$ and $I_{2},$ which is also obtainable from four frames, is given as

$$\begin{matrix} I_{1}+I_{2}=\left[I\left(\mathbf{r};0\right)+I\left(\mathbf{r};\pi/2\right)+I\left(\mathbf{r};\pi\right)+I\left(\mathbf{r};-\pi/2\right)\right]/4 \\ \equiv S, \end{matrix}$$

Combining the product in Eq. and the sum in Eq. , we can solve for $I_{1}$ and $I_{2}$ explicitly as

$$\begin{matrix} I_{1}=\left(S+\sqrt{S^{2}-4P}\right)/2, \\ I_{2}=\left(S-\sqrt{S^{2}-4P}\right)/2. \end{matrix}$$

After all solutions for $I_{1},I_{2},C_{3}$ and $C_{4}$ are obtained, the measured phase is calculated using the definition as

1. **Derivation of $J_{1}\left(\mathbf{r}\right)$ in Eq. (2) in the main text.**

Note that $J_{1}\left(\mathbf{r}\right)$ is the sum of the intensity of the incident field, $I_{1}\left(\mathbf{r}\right)$, and that of the temporal cross-correlation function at zero delays $\tau=0,$ $C_{4}\left(\mathbf{r}\right),$ specifically,

$$\begin{matrix} J_{1}\left(\mathbf{r}\right)=I_{1}\left(\mathbf{r}\right)+C_{4}\left(\mathbf{r}\right) \\ =\iint d^{2}\mathbf{r}_{1}d^{2}\mathbf{r}_{2}\Gamma_{i}\left(\mathbf{r}_{1}-\mathbf{r}_{2}\right)T\left(\mathbf{r}_{1}\right)T^{*}\left(\mathbf{r}_{2}\right)\left[h_{o}\left(\mathbf{r}-\mathbf{r}_{1}\right)+h_{s}\left(\mathbf{r}-\mathbf{r}_{1}\right)\right]h_{o}^{*}\left(\mathbf{r}-\mathbf{r}_{2}\right). \end{matrix}$$

Note that the sum $h_{o}+h_{s}$ is a coherent PSF given by the Fourier transform of the aperture function of the objective of the microscope. This PSF is typically much narrower than $\Gamma_{i}$ and almost zero everywhere except around $\mathbf{r}\approx\mathbf{r}_{1}.$ Ignoring the contribution from terms with $\mathbf{r}\neq\mathbf{r}_{1}$, Eq. simplifies to

$$\begin{matrix} J_{1}\left(\mathbf{r}\right)\approx\iint d^{2}\mathbf{r}_{2}\Gamma_{i}\left(\mathbf{r}-\mathbf{r}_{2}\right)T\left(\mathbf{r}\right)T^{*}\left(\mathbf{r}_{2}\right)h_{o}^{*}\left(\mathbf{r}-\mathbf{r}_{2}\right) \\ =\left\{T\left[Tⓥ_{\mathbf{r}}\left(\Gamma_{i}h_{o}^{*}\right)\right]^{*}\right\}\left(\mathbf{r}\right). \end{matrix}$$

As a side note, computing $J_{1}\left(\mathbf{r}\right)$ now requires only $O\left[N^{2}\log\left(N\right)\right]$ operations using the Fast Fourier Transform (FFT), which is much more effective compared to computing the intensity $I\left(\mathbf{r}\right).$ Therefore, it is more efficient to solve for the transmittance $T\left(\mathbf{r}\right)$ from $J_{1}\left(\mathbf{r}\right)$ instead of from $I\left(\mathbf{r}\right).$

1. **Description of the halo artifact.**

Taking the arguments of Eq. , we have

where is the argument of sample transmittance. It can be seen that when the filtering operation is perfect, i.e. $\left(\Gamma_{i}h_{o}^{*}\right)$ is uniform, the measurement yields the correct phase value, (after trivial constant offset or background subtraction). Essentially, this condition implies perfectly coherent illumination, infinitesimally thin phase annulus and phase ring. Unfortunately, these conditions are almost impossible to achieve in practice as an infinitely thin ring passes no power. At the other extreme, for completely incoherent illumination, we have $\left(\Gamma_{i}h_{o}^{*}\right)=\delta^{\left(2\right)}\left(\mathbf{r}\right),$ the 2D delta-function. As a result, all phase information is lost, i.e., $\arg\left(J_{1}\right)\to0.$ All current commercial PC microscopes fall between these two limits. For such cases, $\arg\left(J_{1}\right)$ is the difference between the true phase and its smoothed out version,$\arg\left[Tⓥ_{\mathbf{r}}\left(\Gamma_{i}h_{o}^{*}\right)\right].$ The phase map, $\arg\left(J_{1}\right)$, therefore, underestimates the true phase by an amount of $\arg\left[Tⓥ_{\mathbf{r}}\left(\Gamma_{i}h_{o}^{*}\right)\right]$. This is the cause of the halo artifacts, which is a well-known phenomenon in phase contrast microscopy [^5^](#_ENREF_5). Figure S1 illustrates the halo and phase-underestimation phenomena. Due to the non-uniform nature of the reference field (red profile), the imaging field only carries high-frequency information of the sample transmission. On the camera plane, these two fields interfere and their phases are subtracted. The net result, denoted by the green profile, has phase values around sharp transitions smaller than that of the background. This phenomenon is called the “halo effect”, commonly known in phase-contrast microscopy [^5^](#_ENREF_5). Also, in the middle of flat regions that are larger than the coherence area, the measured phase is smaller than the correct phase


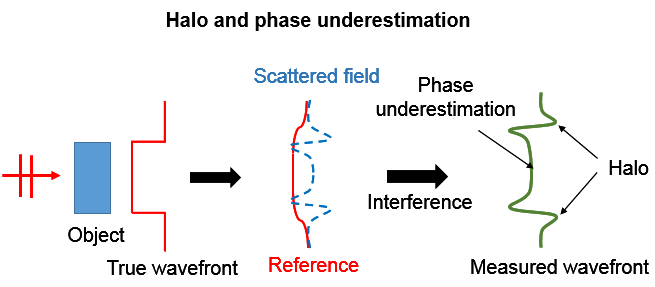


**Figure S1.** Illustration for phase underestimation and halo phenomena.

1. **Solving for the optimized phase and post-processing.**

In order to solve the optimization problem in Eq. (5) in the main text, the Limited memory [Broyden–Fletcher–Goldfarb–Shanno](https://en.wikipedia.org/wiki/BFGS_method)with box constraint (L-BFGS-B) [^6^](#_ENREF_6) algorithm was used. Our source code was developed in Matlab and the call for the L-BFGS-B subroutine was through a Matlab MEX-wrapper written by Stephen Becker (available at <https://github.com/stephenbeckr/L-BFGS-B-C>). At each iteration, only the value of the objective function and its derivative , evaluated at the current estimation , are needed. The trade-off constant is fixed to $\lambda=0.05$. The non-negative constrain is embedded inside the solver to make sure the solution does not have negative phase values with respect to the background. The algorithm is stopped when the updating error is less than a threshold, i.e., or the maximum number of iterations has been reached. Here, the maximum number of iterations is set to 50. However, our experiments showed that very small improvements in reconstruction quality are made after 15-20 iterations. After finding the optimizer we scale it by a scaling factor to match its dynamic range to that of the input image The dynamic range of each image is defined as the difference (in phase) between 2% and 98% percentiles of the values in the image. This normalization step makes the reconstruction more robust to modeling and approximation errors when the quasi-coherent assumption, $I_{1}I_{2}\approx C_{3}C_{4}$, is made.

1. **Calculation of the halo-free phase contrast (hfPC) image.**

Given the halo-free phase map and the halo-free sample transmission obtained by solving the optimization problem, the hfPC intensity image can be computed easily. Practically, a conventional PC optical setup comes with a ring illumination annulus, that is sufficiently thick, to increase the illumination power and, therefore, boost the acquisition signal-to-noise ratio. However, this type of illumination is not necessary in order to compute the hfPC images from the sample transmission $T^{\dagger}\left(\mathbf{r}\right)$. Instead, a pin-hole illumination can be used, i.e., $\tilde{\Gamma}_{i}\left(\mathbf{k}\right)=\delta^{\left(2\right)}\left(\mathbf{k}\right),$ which means only the transverse spatial frequency $\mathbf{k}=0$ propagates through the system. Using Eq. with $n=1$ for the positive PC image, we obtain the positive hfPC image as

Here, the kernels $h_{o}$ and $h_{s}$ can be obtained from their respective Fourier transforms $\tilde{h}_{o}\left(\mathbf{k}\right)$ and $\tilde{h}_{s}\left(\mathbf{k}\right).$ Since the support of the phase ring of the objective needs to be matched the illumination annulus in PC microscopy, we can obtain these function as $\tilde{h}_{o}\left(\mathbf{k}\right)=1\left(\mathbf{k}=\mathbf{0}\right)$ and $\tilde{h}_{s}\left(\mathbf{k}\right)=\Pi\left[k/\left(\beta_{o}NA_{o}\right)\right]-\tilde{h}_{o}\left(\mathbf{k}\right).$ Here, the function $1\left(x\right)$ is the Kronecker delta function, taking the value of 1 of $x=0$ and 0, otherwise.

1. **Halo-removal from micro-pillar images.**

Figure S2 shows the halo-removing results for the different types of square pillars of 10, 20 and 40-µm width, under $20\text{x}$ and 40x magnifications. It can be seen that the correction for the halo artifacts and phase-underestimation is almost perfect up to 20-µm wide pillar at 20x and up to 10-µm wide pillar at 40x magnification. Improvement can be seen for larger pillars as well.


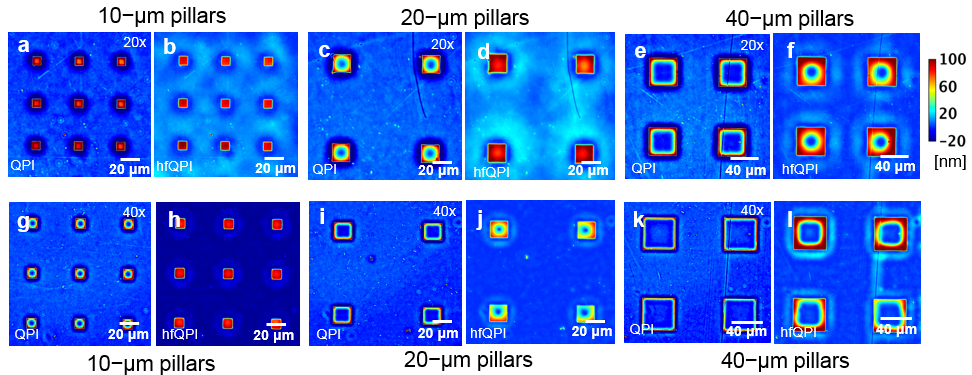


**Figure S2.** **a**-**l,** Raw QPI and hfQPI images of the micropillars of various sizes at different magnifications. It can be seen that the correction performance reduces when the dimension of the object get larger or at higher magnifications. For example, at 20x magnification, 20-µm pillars can be fixed correctly. At 40x magnification, correct images of 10-µm pillars are obtained. Moderate improvement can be observed for larger pillars.

To quantify the amount of improvement, we use a metrics named “contrast ratio” (CR). Figure S3a shows how this ratio is calculated. The ratio is calculated by dividing the area under the height profile (S_1_) through the center of the pillar to the expected area under perfect reconstruction (S_2_). A contrast ratio of 1.0 corresponds to a pillar with no halo. Figure S3b shows a scatter plot for the contrast ratios before and after halo-removal. Improvement in the CR can be observed in all cases i.e. all points lie above the $y=x$ black dash line. However, the improvement is more significant with 20x magnification compared to the 40x magnification.


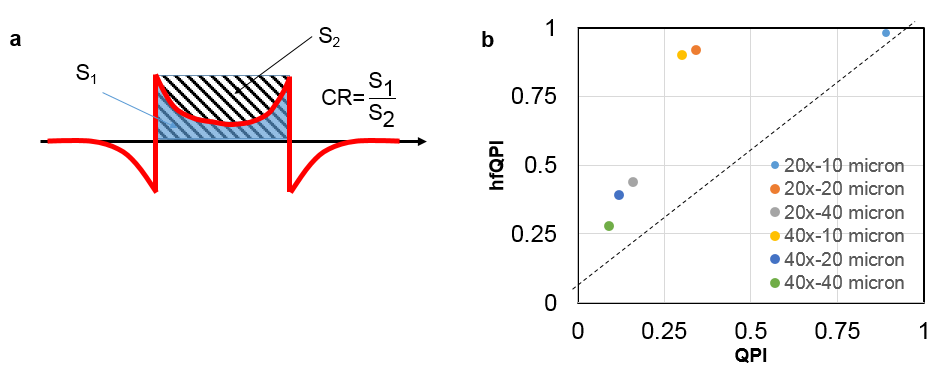


**Figure S3.** **a**, Calculating CR for the pillar sample. The ratio is computed by dividing the area under the central thickness profile of the pillar to the expected thickness profile. **b**, A scatter plot of the CR for various sizes of the pillars at different magnifications evaluated using the original QPI and hfQPI images.

1. **Performance comparison between QPI, tQPI and hfQPI.**

Next, we compare the effects of the halo-removal process using histograms of phase value. Figs. S4a-c show a raw QPI, tQPI, and hfQPI images, respectively. The tQPI image is computed by zeroing all negative phase values in the raw QPI image. Figure S4d shows 256-histograms of the phase value obtained from these images. The maximum value of the histograms are obtained at the 0-phase bin for all of these images due to lots of contribution from the background. The histograms from the raw QPI and the tQPI are identical for positive phase value. The histogram of phase values from the hfQPI image is very close to those from the original QPI image and tQPI image for small positive phase value e.g. [0.0, 0.2] radians. However, more fractions of pixels are distributed towards the larger phase in the hfQPI image than in the QPI or the tQPI image. These values are due to our correction, which boosts the underestimated values of the raw QPI image


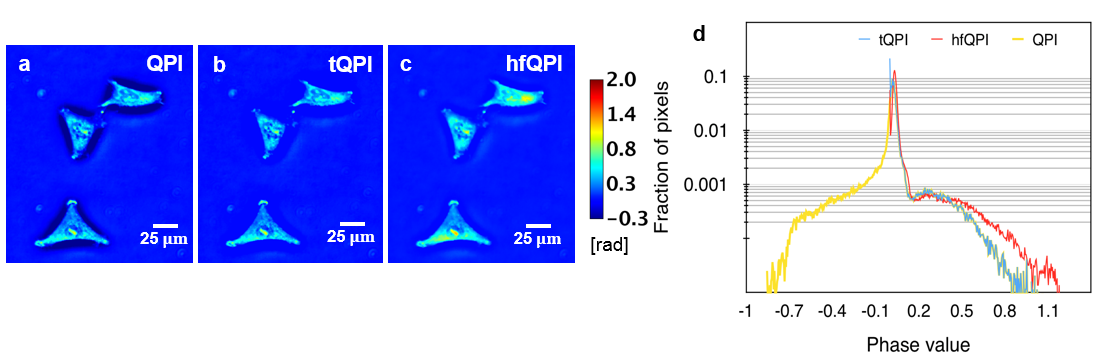
**Figure S4. a**-**c**, QPI, tQPI, and hfQPI images of Hela cells. **d**, Histogram distribution of the phase value in log 10-scale of these images.

1. **Automatic cell segmentation for 20x Hela cell images.**

The cells are automatically segmented from the phase map as follows. Here, we illustrate the process using an hfQPI image of a Hela cell in Fig. S5a. First, measurement noise is removed from the each image by filtering them with a Gaussian’s kernel and a standard deviation of one pixel. Second, the Sobel’s edge detector is applied to find the edges of all cells (Fig. S5b). Third, detected edges are dilated using line structure elements of length 4 at 0 and 90 degrees (Fig. S5c). Finally, holes inside positive regions are filled and regions with less than 3000 pixels are eliminated (Fig. S5d). The final segmentation result is shown in Fig. S5e.


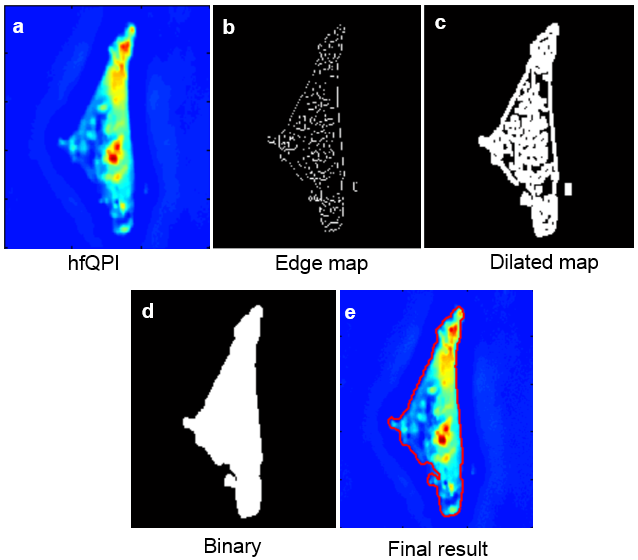


**Figure S5.** **Automatic segmentation diagram. a**, Input hfQPI image. **b**, Edge map obtained by the Canny edge detector. **c**, Dilated edge map. **d**, Binary map of the cell obtained by filling all holes of (**c**). **e**, Final segmentation results obtained by overlaying cell boundaries over the hfQPI image.

More segmentation results are shown in Fig. S6.


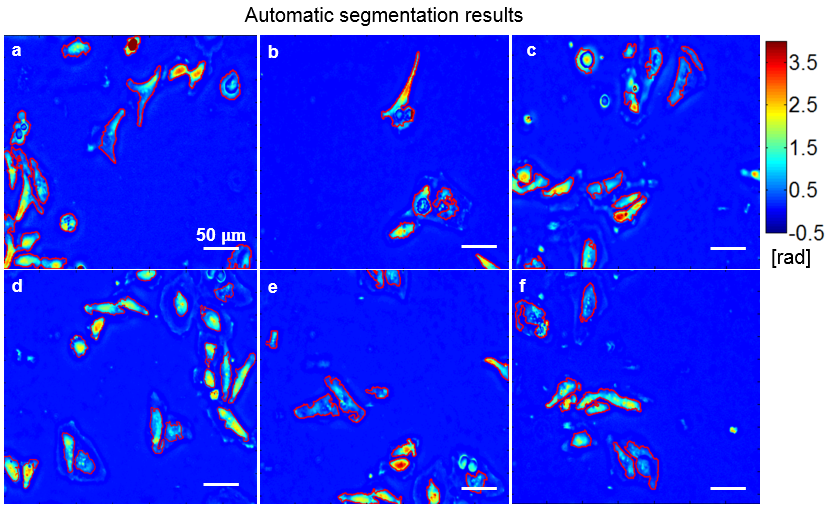


**Figure S6.** **a**-**f,** More results on automatic segmentation of Hela cells. Here, the cell boundaries are overlaid on hfQPI. The same colorbar applies for all images.

**Dry mass from tQPI images and hfQPI images.**


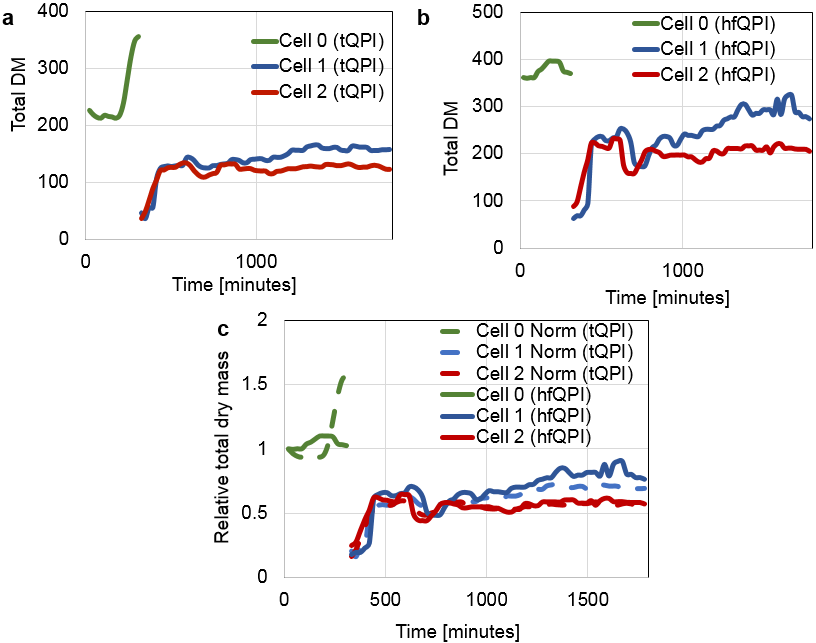


**Figure S7.** **a**, Total dry mass vs. time of a parent Hela cells and its two daughter cells from tQPI images. **b**, Total drymass vs. time of a parent Hela cells and its two daughter cells from the hfQPI image. **c**, Relative dry masses from tQPI images and those from hfQPI images. DM: dry mass.

Absolute values of the total dry mass from tQPI images and hfQPI images are shown in Fig. S7a and Fig. S7b, respectively. It can be seen that at each time point, the absolute dry mass from the hfQPI image is proportional to that from the tQPI images. Therefore, we expect the dry mass from these two techniques to be quite similar to each other, in relative terms. This expectation is verified by Fig. S7c where relative dry masses obtained from two techniques are shown simultaneously. Strong agreement can be observed between these quantities.

**References**

1 Mehta, S. B. & Sheppard, C. J. Using the phase-space imager to analyze partially coherent imaging systems: bright-field, phase contrast, differential interference contrast, differential phase contrast, and spiral phase contrast. *‎J. Mod. Opt.* **57**, 718-739 (2010).

2 Goodman, J. W. Statistical Optics. *New York, Wiley-Interscience* **1,** (1985).

3 Sheppard, C. J. & Mehta, S. B. in *Frontiers in Optics.* FTu2E. 1 (Optical Society of America).

4 Wang, Z. *et al.* Spatial light interference microscopy (SLIM). *Opt. Exp.* **19,** (2011).

5 Zernike, F. How I discovered phase contrast. *Science* **121**, 345-349 (1955).

6 Zhu, C., Byrd, R. H., Lu, P. & Nocedal, J. Algorithm 778: L-BFGS-B: Fortran subroutines for large-scale bound-constrained optimization. *ACM Transactions on Mathematical Software (TOMS)* **23**, 550-560 (1997).
